# High CCR6 expression increases the risk of pediatric Langerhans cell histiocytosis

**DOI:** 10.1097/BS9.0000000000000224

**Published:** 2025-04-16

**Authors:** Xingfeng Yao, Yutian Zheng, Jiasi Xia, Meng Zhang, Wentao Zheng, Rui Zhang, Yaqian Wu, Lejian He, Honggang Liu

**Affiliations:** a Department of Pathology, Beijing Tongren Hospital, Capital Medical University, Beijing Key Laboratory of Head and Neck Molecular Diagnostic Pathology, Beijing, China; b Department of Pathology, Beijing Children’s Hospital, Capital Medical University, National Center for Children’s Health (NCCH), Beijing, China; c Beijing Key Laboratory of Pediatric Hematology Oncology, National Key Discipline of Pediatrics, Key Laboratory of Major Diseases in Children, Ministry of Education, Hematology Oncology Center, National Center for Children’s Health, Beijing Children’s Hospital, Capital Medical University, Beijing, China

**Keywords:** BRAF V600E, CCR6, CCR7, Langerhans cell histiocytosis, MAP2K1

## Abstract

Langerhans cell histiocytosis (LCH) is a rare disorder that primarily affects children. Considering the intricate clinical presentation of this disease, the identification of specific biomarkers associated with susceptibility to LCH is essential for timely diagnosis and risk stratification. In this study, we examined the skin specimens from pediatric patients with LCH using RNAscope, immunohistochemistry, and sequencing techniques. We observed a notable correlation between elevated CCR6 expression in pathological tissues and LCH risk classification. Therefore, CCR6 expression may serve as an independent predictor of risk in clinical cases of LCH. Furthermore, the frequency of *BRAF* V600E mutations correlated with risk stratification. We discovered new mutations—H119Y and R108Q—in *MAP2K1* in specimens with *BRAF* V600E mutations. Moreover, CCR6-positive tumors may exhibit an enhanced recruitment of lymphocytes expressing high CCR7 levels.

## 1. INTRODUCTION

Langerhans cell histiocytosis (LCH) is a rare histiocytic disorder with ongoing debate regarding its classification as an inflammatory or neoplastic disease. LCH was defined as a neoplastic lesion of myeloid origin following the discovery of the *BRAF* V600E mutation, a classification that persisted until 2010.^1^ The annual incidence of LCH is 0.5 to 5.4 cases per 100,000 individuals in China, with a predominance in males. Although LCH can occur in individuals of all age groups, it is more commonly diagnosed in children and has a relatively low incidence in adults.^2,3^ LCH is further categorized into single-system (SS-LCH) and multi-system (MS-LCH) forms. Additionally, based on the extent of organ involvement, LCH can be classified into single- and multi-organ types. MS-LCH is frequently observed in the skin, bones, lungs, and pituitary gland. The management of MS-LCH involves a higher risk, whereas SS-LCH, such as skeletal or skin lesions in children, often resolves spontaneously. Severe LCH cases can present with life-threatening systemic multi-organ or multi-system lesions.^4^ A staging system for LCH remains lacking, and patients with LCH are classified into low- and high-risk groups based on the presence or absence of high-risk organ involvement. The low-risk group encompasses individuals without high-risk organ involvement, typically involving the skin, bones, lymph nodes, and thymus. Conversely, the high-risk group encompasses individuals with high-risk organ involvement, such as the liver, spleen, blood, and central nervous system (CNS).^5^

The clinical presentation of LCH is heterogeneous owing to its overlapping features with common tissue lesions. It can manifest as a self-limiting rash or bone destruction or can lead to life-threatening multi-organ damage. This heterogeneity makes visual recognition of LCH challenging. Therefore, once LCH is suspected, the diagnosis should be confirmed by considering a combination of clinical manifestations, histopathology, and other relevant factors.^4,6^ In approximately 50% of children, a head and neck rash—a common symptom of this inflammatory neoplastic disease originating in the medulla—presents early in the disease. Furthermore, the liver commonly exhibits sclerosing cholangitis and splenic involvement as the characteristic manifestations. CNS lesions associated with LCH can be categorized into two main types: those resulting from Langerhans cell infiltration, often presenting as pituitary infiltration, and neurodegenerative lesions, which include ataxia and dysphagia.^4,7,8^ Although LCH presents with a range of clinical symptoms and imaging manifestations, the most reliable way to confirm the diagnosis is pathological testing. This involves a preliminary diagnosis based on microscopic observation using hematoxylin and eosin (HE) staining as well as immunophenotyping, wherein a positive CD207 (Langerin) or CD1a antigen is sufficient for diagnosis.^9^ Additionally, electron microscopy can be used to observe the presence of Birbeck granules in lesion cells to confirm the diagnosis.^10^

The ongoing investigations into additional specific markers associated with the etiology and progression of LCH have extended beyond CD1a and CD207. LCH is an inflammatory neoplastic disorder, and the inflammatory milieu directly affects disease progression. Chemokines, particularly those belonging to the CC and CXC families, play crucial roles in the recruitment of cells during inflammation, directing the migration of leukocytes and influencing the function of infiltrating immune cells.^11^ The human *CCR6* (also referred to as *CD196*) gene is located at chromosomal position 6Q27 and comprises four exons, encompassing a total of 1235 bases. *CCR6* encodes a protein of a molecular weight of 42.5 kDa consisting of 374 amino acids. CCR6 functions as a receptor for CCL20, which is present on the surface of various immune cells, including B lymphocytes, T lymphocytes, plasma cells, natural killer cells, dendritic cells, and neutrophils. CCR6 is highly expressed in pro-inflammatory and Treg cells and plays a crucial role in the migration and localization of immune cells.^12^ CCR6 upregulation is often observed in a diverse array of inflammation-related disorders. It is a promising viable therapeutic target for the development of antibody-based treatments for inflammatory ailments. In the context of LCH, an accumulation of CCR6 and CCR7 within affected tissues suggests that CCR7 may play a role in recruiting CCR6 from non-lymphoid organs, such as the skin and bone, to the lymphoid tissues for enrichment. Consequently, CCR6 has emerged as a potential target for LCH diagnosis and treatment.^13,14^

Rapid advances in molecular pathology in recent years have significantly enhanced the diagnosis and treatment of several diseases. The identification of the *BRAF* V600E mutation has not only clarified the etiology of LCH but has also paved the way for exploring the signaling pathway associated with the disease. Previous research has demonstrated a strong correlation between aberrant activation of the MAPK pathway and LCH development, affecting approximately 85% of pediatric patients with the disease.^15,16^ Specifically, *BRAF* V600E mutations are observed in approximately 60% of pediatric LCH cases.^17,18^ The *BRAF* V600E mutation, which manifests at various stages of cellular development, occurs at different developmental stages in hematopoietic cells, indirectly affecting the clinical manifestations and typing. If the mutation occurs at the bone marrow stem progenitor cell stage, the clinical manifestations are typically classified as multisystemic low-risk. If the mutation occurs only at the Langerhans cell stage, the clinical manifestation is considered a single-cell low-risk. Nevertheless, no study has demonstrated a definitive correlation between the *BRAF* V600E mutation and patient risk.

The present study involved 110 pediatric patients admitted to Beijing Children’s Hospital with the confirmed diagnosis of LCH using routine HE staining, immunohistochemistry, and electron microscopy of pathological tissue samples. We assessed CCR6 and CCR7 expression in these samples using RNAscope and immunohistochemistry. Our findings, for the first time, revealed a positive correlation between CCR6 expression and the risk grouping of children, indicating its potential as a prognostic marker. Furthermore, a positive correlation was observed between the CCR6 and CCR7 expressions in these samples. We also employed polymerase chain reaction (PCR) and sequencing to compare and statistically analyze *BRAF* and *MAP2K1* mutation sites in all samples. Our in-depth analyses revealed a correlation between *BRAF* V600E mutations and risk grouping in children, as the *BRAF* V600E mutation was detected in all CCR6-positive tissues. Furthermore, the identification of mutations in *MAP2K1* in *BRAF*-mutated tissues further supports the significance of the MAP2K1–BRAF signaling pathway in the development of LCH.

## 2. MATERIALS AND METHODS

### 2.1. Case selection and tissue handling

Tissue samples and clinical information of 110 diagnosed Chinese pediatric patients with LCH (63 males and 47 females; median age, 3.7 [range, 0.4–14.3] years) presented to the Beijing Children’s Hospital, Capital Medical University, National Center for Children’s Health between 2020 and 2023 were obtained. Consent from the guardians was obtained before the collection and use of all skin tissue specimens. The diagnosis of LCH was confirmed based on the histopathological findings in biopsy samples and the aggregation of CD1a-positive histiocytes and/or Langerin immunohistochemical staining in all cases. Patients with LCH were classified into 3 groups: MS-LCH, CNS-LCH, and SS-LCH. MS-LCH was defined as the presence of lesions in several organs, whereas SS-LCH was defined as lesions in only 1 organ. The MS-LCH, CNS-LCH, and SS-LCH groups consisted of 9, 26, and 75 patients, respectively. Patients with MS-LCH were further classified based on liver, spleen, and hematopoietic or CNS involvement according to the criteria of the Histiocyte Society.^19,20^ Tissues for microscopic examination were preserved in neutral-buffered 10% formalin, processed in a routine manner, sliced into 5-μm-thick sections, and stained with HE.

### 2.2. Diagnostic criteria and risk stratification for LCH

The diagnosis of LCH was confirmed using the following steps, preliminary diagnosis: LCH was clinically suspected based on clinical manifestations and imaging examinations; pathological tissue acquisition: the surgical method to obtain the most accessible and representative lesions, such as biopsy of the skin rash or ring drilling, scraping or puncturing of bone lesions, or excision or puncture of organ or systemic lesions, was selected according to the location and size of the lesion; pathological examination: the tissues were embedded in paraffin and subjected to HE and immunohistochemical staining; histological evaluation: the diagnosis of LCH was confirmed when the tissue morphology showed foci with mucinous neoplastic Langerhans cells, with CD1a- and Langerin-positive tumor cells; otherwise, LCH was excluded, and biopsies were taken to re-examine the suspected cases that were not diagnosed conclusively. The risk of missed and misdiagnosed LCH is specifically high when it involves the lymph nodes. The diagnosis is confirmed if partial destruction of the lymph node structure is observed, particularly when the lesion involves the lymphatic sinus. In cases of atypical lesions, differentiation from reactive hyperplasia is necessary, where Langerhans cells are focally or patchily distributed in LCH, often accompanied by scattered or eosinophilic infiltration. In reactive hyperplasia, lymph node structures are usually intact with scattered Langerhans cells. This should be considered alongside clinical evidence of multifocal, multisystemic, organ involvement to facilitate LCH diagnosis.

Risk stratification for LCH is performed in 2 steps. A positive *BRAF* V600E mutation result is essential to guide the use of targeted therapies in symptomatic or rapidly progressing children, initially determined based on *BRAF* V600E immunohistochemistry and secondarily confirmed via mutation testing. A comprehensive evaluation is then performed using abdominal ultrasonography, liver function, lung function, CNS imaging, lung imaging, bone imaging, bone marrow aspiration, and other relevant tests. Based on the extent of involvement at diagnosis, the patients were classified into SS-LCH and MS-LCH. SS-LCH is defined as the involvement of only one organ or system, whereas MS-LCH is defined as the involvement of 2 or more organs or systems with or without risk organs, including the liver, spleen, and hematological system. CNS-risk lesions refer to bone lesions in the mastoid, sphenoid, orbit, clivus, or temporal bone and involvement of the oral cavity, ear, or eye, which indicate an increased risk of developing neurodegenerative CNS-LCH.^20–22^

### 2.3. DNA extraction and quantitative PCR

To remove the paraffin, 1 mL of xylene was added to the labeled tube containing the specimen and gently shaken on a rocker for 1 minute. The samples were then pelleted by spinning at 13,000 rpm in a microcentrifuge for 3 minutes. Next, the xylene supernatant was carefully removed and discarded in a polypropylene tube. This xylene wash was repeated until the paraffin was completely dissolved. Thereafter, ethanol rehydration was performed by air-drying the pellet for 5 minutes, ensuring not to overdry. The samples were then incubated with 100 μL lysis buffer at 56°C in a heating block until the tissue was fully dissolved. An equal volume of buffer-saturated phenol was added to clean up the protein, and DNA was quantified with A260/A280 using a NanoDrop spectrophotometer. The universal primers M13F and M13R were applied for *BRAF* and *MAP2K1*.

### 2.4. Immunohistochemistry

Immunohistochemical analysis was performed using 3-µm sections, obtained from the formalin-fixed paraffin-embedded (FFPE) specimen on an automated staining system (Leica BOND MAX, Australia) with antibodies against CCR6 (1:500; ab227036; Abcam, Cambridge, England) and CCR7 (1:100; ab253187; Abcam) following the manufacturer’s instructions. Two blinded pathologists independently evaluated the immunohistochemical slides and scored the expression of target proteins. CCR6 expression was scored positive if cytoplasmic staining was observed in LCH cells. The intensity of CCR6 and CCR7 expression was graded as “−” for no cytoplasmic staining, “+” for weak cytoplasmic staining, “±” for strong staining in <30% of cancer cells, “++” for strong staining in >30% and <50% of cancer cells, and “+++” for strong staining in >50% of cancer cells. Notably, staining in ≥10% area was considered positive for each specimen. CCR6^low^ indicates “−” and “+,” CCR6^med^ indicates “+ −” and “++,” and CCR6^hi^ indicates “+++.”

### 2.5. RNAscope

RNAscope (Advanced Cell Diagnostics, Hayward, California) was performed using probes targeting CCR6 and CCR7 on TMA slides, following the manufacturer’s protocol. The RNAscope procedure included the following steps: first, the TMA tissue sections were deparaffinized and sequentially subjected to pretreatment 1 (10 minutes, room temperature), pretreatment 2 (boiling for 20 minutes), and pretreatment 3 (30 minutes, 40°C). Second, slides were hybridized with target probes and incubated in a HybEZ oven for 2 hours at 40°C. Third, the signals were amplified and generated using an RNAscope 2.0 HD Reagent Kit-BROWN. CCR6 and CCR7 expression was scored as positive if cytoplasmic staining was observed in LCH cells. Two blinded pathologists independently scored the expression of target proteins. The intensity of CCR6 and CCR7 expression was graded as “−” for no cytoplasmic staining, “+” for weak cytoplasmic staining, “±” for strong staining in <30% of cancer cells, “++” for strong staining in >30% and <50% of cancer cells, and “+++” for strong staining in >50% of cancer cells. Notably, staining in ≥10% area was considered positive for each specimen. CCR6^low^ indicates “−” and “+,” CCR6^med^ indicates “+ −” and “++,” and CCR6^hi^ indicates “+++.”

### 2.6. Transmission electron microscopy

Selected formalin-fixed tissue samples obtained from all children with LCH were submitted to the transmission electron microscopy laboratory in Beijing, China. The samples were fixed with Karnovsky fixative, followed by 2 washes in 0.2 M sodium cacodylate, and post-fixation treatment with 2% osmium tetroxide reduced with 2.5% potassium ferrocyanide. The tissues were then washed in 0.2 M sodium cacodylate and dehydrated through a graded ethanol series before infiltration and embedding in Spurr formulation of Eponate 12 epoxy resin (Ted Pella, Redding, California). The samples were sliced into thick sections, mounted on glass slides, and stained with toluidine blue O. Next, the samples were sliced into thin sections, mounted on 150 mesh copper grids, and stained with 4% uranyl acetate in 75% ethanol and Reynold lead citrate. Finally, the sections were examined using a Zeiss 10 C and Zeiss 906E transmission electron microscope (Zeiss Electron Microscopy, Thornwood, New York), respectively.

### 2.7. Statistical analysis

SPSS (version 26; SPSS Inc., Chicago, Illinois) was used for statistical analysis. The data are expressed as the mean ± standard deviation (SD), and the data from specific experiments were compared using 1-way analysis of variance or Student *t* test or χ^2^ test. GraphPad Prism 6 software (GraphPad Software, San Diego, California) was used for visualization and to prepare figures and charts. Chi-square analysis was used to analyze the differential CCR6 and CCR7 expression in LCH specimen. All experiments were repeated at least 3 times, with consistent results. *p* < 0.05 was considered statistically significant.

## 3. RESULTS

### 3.1. Participants

**Figure 1.**
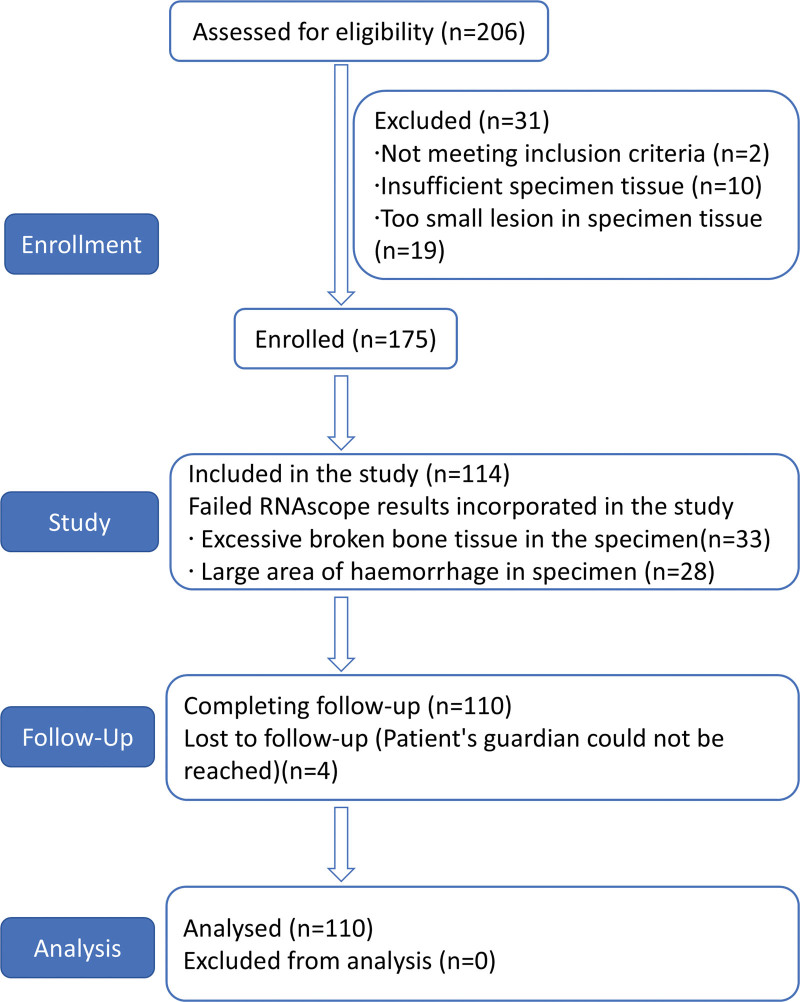
Participants section flow diagram.

In this study, 206 children with a confirmed diagnosis of LCH were initially enrolled based on their year of consultation and age. Following screening based on the completeness of the clinical information and adequacy of the corresponding pathological tissue specimens, 175 children and their pathological specimens were included in this study. Furthermore, 61 cases were excluded due to inadequate tissue quality and experimental data, and 4 cases were lost to follow-up, resulting in a total of 110 children with valid pathological test data. Figure 1 illustrates the flowchart of participant recruitment. Table 1 presents the main baseline characteristics of the enrolled patients.

**Table 1:** Basic information, risk stratification, and organ involvement.

Characteristic	All patients (cases [%])
Total	110
Gender, n (%)	
Male	63 (57.3)
Female	47 (42.7)
Age, n (%)	
≤3	59 (53.6)
>3	51 (46.4)
Risk stratification	
BRAF V600E, n (%)	
Positive	69 (62.7)
Negative	41 (37.3)
Organ involvement, n (%)	
SS-LCH, n (%)	75 (68.2)
Skin	27 (24.5)
Face	9 (8.2)
External auditory meatus	4 (3.6)
Head	2 (1.8)
Neck	3 (2.7)
Trunk	9 (8.2)
Bone	34 (30.9)
Parietal	7 (6.4)
Frontal	5 (4.5)
Humeral	1 (0.9)
Iliac	4 (3.6)
Zygomatic	2 (1.8)
Occipital	4 (3.6)
Cervical	11 (10)
Lung	5 (4.5)
Bronchial tube	1 (0.9)
Mediastinum	5 (4.5)
Lymph nodes	3 (2.7)
Cervical	2 (1.8)
Mediastinal	1 (0.9)
MS-LCH, n (%)	9(8.2)
Cervical lymph nodes, liver, spleen	2 (1.8)
Thyroid, liver, spleen, blood	1 (0.9)
Mediastinal lymph nodes, lungs, liver, parietal bone	1 (0.9)
Mediastinal lymph nodes, lung, liver, spleen	1 (0.9)
Liver, spleen, blood	1 (0.9)
Liver, lung, skin	1 (0.9)
Facial skin, external auditory canal	2 (0.9)
CNS-LCH, n (%)	26 (23.6)
Maxillary bone	7 (6.4)
Cranial bone	10 (9.1)
Temporal bone	4 (3.6)
Orbit	4 (3.6)
Bone marrow	1 (0.9)

CNS-LCH = central nervous system Langerhans cell histiocytosis, MS-LCH = multi-system Langerhans cell histiocytosis, SS-LCH = single-system Langerhans cell histiocytosis.

### 3.2. Clinical LCH diagnosis

**Figure 2.**
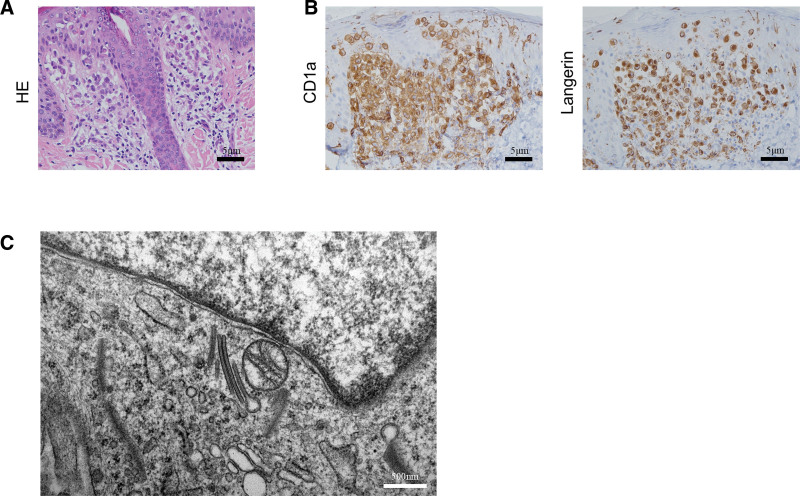
Clinical LCH diagnostic basis. (A) Representative image of HE staining of pathological tissue from the LCH child. (B) Representative image of immunohistochemistry (IHC) staining results of the CD1a and Langerin positivity on pathological tissue of the LCH child. (C) Representative diagram of typical structures shown by electron microscopy of pathological tissue in the LCH child. HE = hematoxylin and eosin, LCH = Langerhans cell histiocytosis.

In this study, 110 children diagnosed with LCH were examined via HE, immunohistochemistry, and electron microscopy. The age of the children ranged from 2 months and 4 days to 12 years, with 53.6% being ≤3 years. The male-to-female ratio was 1:0.65. Children were categorized into the SS-LCH and MS-LCH groups based on the presence or absence of risk-organ involvement. The MS-LCH group was further subdivided based on hepatic, splenic, and hematological or CNS involvement (CNS-LCH). The statistical analysis of the clinical data revealed 75 and 35 patients in the SS-LCH and high-risk groups, respectively, with 9 and 26 patients from the high-risk group further categorized in the MS-LCH and CNS-LCH groups, respectively (Table 2). The pathological evaluation of LCH involved the initial diagnosis via HE staining of skin tissues, revealing typical LCH morphology under light microscopy, abundant light-colored cytoplasm, kidney- and coffee-bean-shaped nuclei, and well-defined nuclear grooves (91/110). Additionally, infiltration of multiple inflammatory cell, such as lymphocytes, eosinophils, and multinucleated giant cells, was observed in the background (70/110) (**Fig. 2A**). Next, CD1a and Langerin (CD207) antigen expression of tissues detected using immunohistochemistry revealed their localization in the cell membrane and cytoplasm, with frequent co-expression (57/110) (**Fig. 2B**). The final diagnosis was confirmed using electron microscopy of the Langerhans granules. Ultrathin sections were microscopically assessed to determine the presence of Birkbeck granules (also known as Langerhans cell granules or X-bodies). These granules were observed in longitudinal sections as straight or curved rod-like membrane structures, approximately 40 nm wide, in all 110 samples. Surrounded by 2 membranes on either side, these particles exhibited a pentahedral structure consisting of a longitudinal row of septa in the center, resembling a zipper-like arrangement with a lateral spacing of 6 to 7 nm (**Fig. 2C**). These findings align with the typical descriptions of Langerhans particles in literature.

**Table 2:** Statistical analysis of correlation between clinicopathological information and CCR6 protein levels in LCH tissue microarray.

Variable^①^	Number (n = 110)	CCR6^low^ (n = 59)	CCR6^med^ (n = 33)	CCR6^hi^ (n = 18)	χ^2^	*P*
Gender					3.825	0.306
Male	63	30	21	12		
Female	47	29	12	6		
Age, y					2.955	0.465
≤3	59	32	16	11		
>3	51	27	17	7		
Group					18.776	0.009*
SS-LCH	75	48	18	9		
MS-LCH	9	2	5	2		
CNS-LCH	26	9	10	7		

CNS-LCH = central nervous system Langerhans cell histiocytosis, MS-LCH = multi-system Langerhans cell histiocytosis, SS-LCH = single-system Langerhans cell histiocytosis.

①The intensity of CCR6 expression was graded as “–” no cytoplasmic staining, “+” weak cytoplasmic staining; “±” strong staining in <30% of cancer cells, “++” strong staining in more than 30% and less than 50% of cancer cells, “+++” strong staining in more than 50% of cancer cells. Ten percent or more of the stained area positive is considered positive for each specimen. CCR6^low^ includes “−” and “+,” CCR6^med^ includes “+ −” and “++,” CCR6^hi^ includes “+++.”

* *p <* 0.01.

### 3.3. High CCR6 expression positively correlates with high risk in pediatric LCH

**Figure 3.**
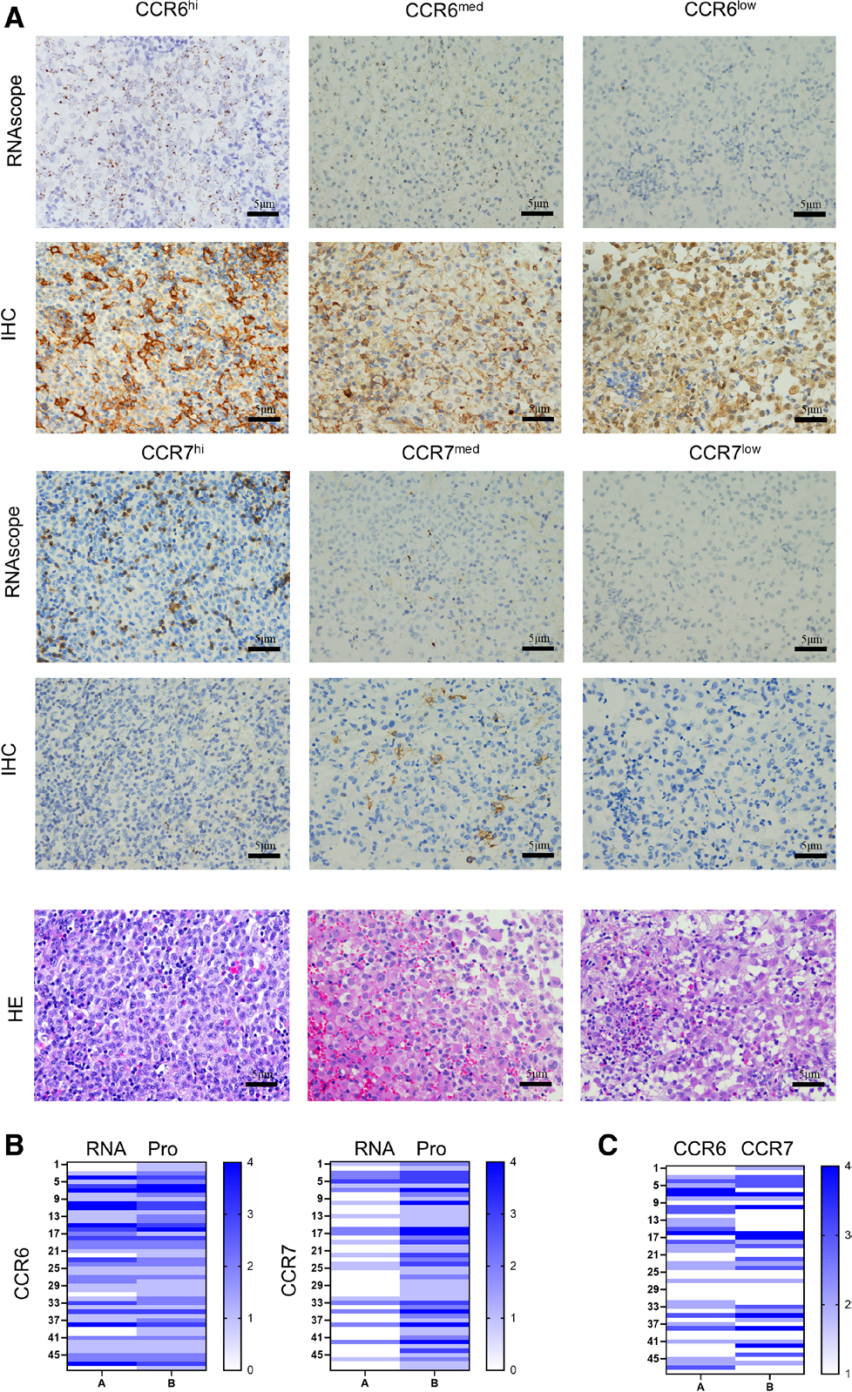
Positive correlation between CCR6 and CCR7 expression in skin tissues of children with LCH. (A) Positive correlation between CCR6 and CCR7 expression in skin tissues of children with LCH. (B) Heatmap of RNA and protein content of CCR6 and CCR7 from pathological tissues of 48 children with LCH. (C) Correlation heat map of CCR6 and CCR7 protein level in pathological tissues of 48 children with LCH. HE = hematoxylin and eosin, LCH = Langerhans cell histiocytosis.

LCH is characterized by inflammatory tumors and a well-established involvement of chemokines in the development of inflammation. Previous studies have reported elevated CCR6 and CCR7 expression in tumor neoplasms. However, the extent to which CCR6 and CCR7 expression in LCH correlates with the risk in pediatric patients remains unexplored. Consequently, we performed RNAscope detection and immunohistochemical staining of tissue sections to determine CCR6 and CCR7 expression (**Fig. 3A**). We observed a significant positive correlation between CCR6 and CCR7 RNA expression in tissues and their respective protein levels (**Fig. 3B**). These results validate the accuracy and reliability of the test data. Additionally, a comparative analysis of CCR6 and CCR7 levels in the same samples revealed a positive correlation between CCR6 and CCR7 expressions (**Fig. 3C**; Table 3). Specifically, samples with high CCR6 expression also exhibited relatively high CCR7 expression, whereas those with low CCR6 expression exhibited low CCR7 expression. Additional statistical analyses, along with clinicopathological indicators, demonstrated a positive correlation between CCR6 levels and the risk groupings of children, as well as with disease progression or recurrence. Specifically, children with higher CCR6 expression exhibited a comparatively elevated risk of developing LCH and an increased likelihood of disease recurrence (Tables 2, 4).

**Table 3:** Statistical analysis of correlation between clinicopathological information, CCR6 and CCR7 protein levels in LCH tissue microarray.

Variables^②^(n = 48)	Number	CCR6^low^ (n = 21)	CCR6^med^ (n = 24)	CCR6^hi^ (n = 3)	χ^2^	*P*
CCR7^low^	21	15	4	2	17.188	0.002*
CCR7^med^	22	5	17	0		
CCR7^hi^	5	1	3	1		

LCH = Langerhans cell histiocytosis.

②CCR6^low^ includes “−” and “+,” CCR6^med^ includes “+ −” and “++,” CCR6^hi^ includes “+++.” CCR7^low^ includes “−” and “+,” CCR7^med^ includes “+ −” and “++,” CCR7^hi^ includes “+++.”

* *p <* 0.01.

**Table 4:** Statistical analysis of correlation between CCR6 protein level and disease progression, relapse in LCH tissue microarray.

Variables^③^	Number (n = 110)	CCR6^low^ (n = 59)	CCR6^med^ (n = 33)	CCR6^hi^ (n = 18)	χ^2^	*P*
Progression					15.833	0.003*
No progress	95	56	28	11		
Relapse	15	3	5	7		

LCH = Langerhans cell histiocytosis.

③CCR6^low^ includes “−” and “+,” CCR6^med^ includes “+ −” and “++,” CCR6^hi^ includes “+++.”

* *p <* 0.01.

**Table 5:** Statistical analysis of correlation between BRAF V600E mutations and risk group.

Variables	Number (n = 110)	BRAF V600E^-^(n = 41)	BRAF V600E^+^ (n = 69)	χ^2^	*P*
Group				1.871	0.368
SS-LCH	75	31	44		
MS-LCH	9	2	7		
CNS-LCH	26	8	18		
Progression					
No progress	95	40	55	6.959	0.009*
Relapse	15	1	14		

CNS-LCH = central nervous system Langerhans cell histiocytosis, MS-LCH = multi-system Langerhans cell histiocytosis, SS-LCH = single-system Langerhans cell histiocytosis.

* *p <* 0.01.

### 3.4. BRAF V600E samples from children with LCH partially exhibit MAP2K1 mutations

**Figure 4.**
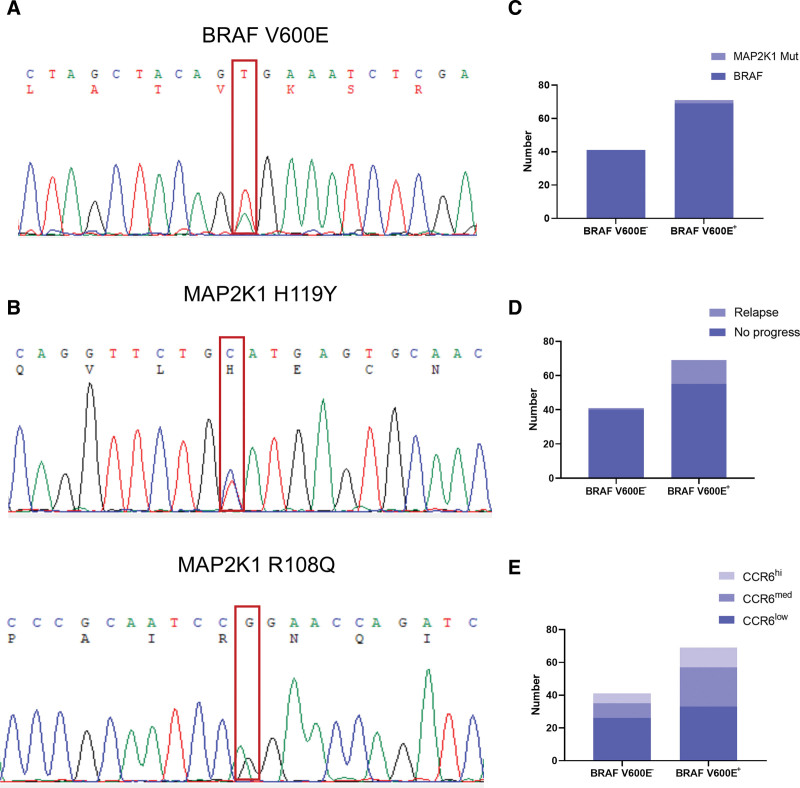
BRAF V600E mutation samples from children with LCH partially have MAP2K1 mutations. (A) Sanger sequencing shows BRAF V600E mutation. (B) Sanger sequencing shows MAP2K1 H119Y and MAP2K1 R108Q mutation. (C) MAP2K1 H119Y and MAP2K1 R108Q mutation all occurred in BRAF V600E mutant samples. (D) Statistical diagram of BRAF V600E mutation and disease progression in children with LCH. (E) Statistical plot of correlation between BRAF V600E mutation and CCR6 protein level content in children with LCH. LCH = Langerhans cell histiocytosis.

LCH is characterized by *MAPK* mutations, with *BRAF* V600E being the most prevalent. This mutation is closely associated with the risk of LCH recurrence. In this study, DNA extracted from paraffin-embedded tissues from a cohort of 110 children was subjected to sequencing analysis targeting *BRAF* and other genes known to be susceptible to mutations in the MAPK signaling pathway, namely MAP2K1. Notably, 69 patients (62.7%) of the cohort exhibited *BRAF* V600E mutations (**Fig. 4A**), with *MAP2K1* mutations in 2 patients among these. The specific *MAP2K1* mutations were C.355C>T(H119Y) and C.323G>A(R108Q) in the exon 3 (**Fig. 4B, C**). Statistical analyses were conducted on the mutation loci and corresponding specimens from affected children to examine disease progression and recurrence. The findings revealed mutations at the *BRAF* V600E locus in 93.3% (14/15) of the children with LCH disease progression or recurrence. In contrast, among the 95 children without disease progression, approximately 57.9% (55/95) displayed *BRAF* V600E mutations (**Fig. 4D, Table 5**). These results indicated a correlation between LCH disease progression and *BRAF* V600E mutation, despite a lack of data supporting a significant correlation between CCR6 expression and *BRAF* V600E mutation. The proportion of tissues with higher CCR6 levels was relatively greater in the *BRAF* V600E-mutated group than in the unmutated group (**Fig. 4E**). Therefore, the *BRAF* V600E mutation may contribute to CCR6 expression.

## 4. DISCUSSION

LCH is distinguished in pediatric oncology because of its diverse clinical presentations, unique histopathological features, immunophenotypes, and ultrastructural properties, which make the diagnostic process more intricate. In addition to conventional HE staining and immunohistochemistry, meticulous electron microscopy is essential. The advent of genetic testing techniques has provided deeper insights into gene expression profiles associated with LCH. In this study, we reported the pathological examination and *BRAF* mutation detection results of 110 pediatric patients with LCH admitted to Beijing Children’s Hospital. CCR6 RNA levels in the tissues were detected using RNAscope. Furthermore, the DNA extracted from these tissues and a loss-of-function of MAPK signaling revealed that 80% of the patients exhibited *BRAF* mutations, with 2 patients exhibiting *MAP2K1* mutations. Additionally, the *BRAF* V600E mutation was associated with an increased risk. Importantly, *BRAF* V600E-related symptoms gradually increased with an increase in CCR6 expression, indicating a positive correlation between high CCR6 and the *BRAF* V600E mutation.

*BRAF*, a proto-oncogene encoding a *RAF* family serine/threonine protein kinase, is expressed in various cancers. *BRAF* affects cell division, differentiation, and secretion by regulating the MAPK/ERK signaling pathway.^20^ Since its discovery in 2002, *BRAF* mutations have been reported in melanoma, non-Hodgkin lymphoma, colorectal cancer, thyroid cancer, and other cancer types.^23,24^ These mutations are classified into types I to III based on their location and functional changes, with type I mutation, V600E, being the most frequent. This mutation strongly activates *BRAF* kinase activity, leading to constitutive activation of the MAPK pathway and subsequent activation of MEK1 and MEK2 proteins, thereby promoting cell proliferation and inhibiting apoptosis.^25^ As early as 2010, LCH was classified as a neoplastic disorder based on the *BRAF* V600E mutation. Since then, the role of this mutation in LCH has been increasingly reported. Although no studies have demonstrated an association between this mutation and specific clinical risk groups, it has been associated with an increased risk of recurrence.^26–28^ Moreover, this mutation has also been reported in immune cell subsets in the tumor microenvironment, such as circulating CD11C and CD14-positive cells and CD34-positive bone marrow hematopoietic progenitor cells, in LCH patients with active and high risk.^29^ However, reports on the correlation between the *BRAF* V600E mutation in LCH and the development or progression of inflammation remain lacking. LCH is an inflammatory disease, and the secretion of chemokines into the environment is closely related to disease progression. By detecting the *BRAF* V600E mutation and CCR6 levels in LCH samples, we initially observed a correlation between the 2, prompting us to investigate the mechanism by which the *BRAF* mutation contributes to the development of the inflammatory environment in LCH.

Chemokines and their receptors are of great significance in maintaining homeostasis in the body. Their uncontrolled activity can lead to chronic inflammation and autoimmune diseases. G protein-coupled receptors are one of the most abundant receptors in the human genome involved in the transduction of approximately 80% of known signals transmitted through the cell membrane. As a receptor of the chemokine CCL20, CCR6 is selectively expressed in immature dendritic cells and memory T cells. By binding to CCL20, CCR6 recruits IL-17-secreting inflammatory cells into the inflammatory environment.^30^ IL-17 is secreted by a highly heterogeneous cell population, which can be classified into Th17, Th22, and T117 subpopulations and unclassified or intermediate populations.^31–33^ Th17 has been reported as a potential therapeutic factor in LCH-related bone destruction. Additionally, the promoting effect of IL-17 on LCH has also been confirmed.^31,34^ CCR6 plays an important role in various diseases, such as autoimmune encephalitis and rheumatoid arthritis.^35^ However, reports on the correlation among CCR6 levels, LCH risk, and disease progression are limited. In this study, we combined disease progression and *BRAF* gene mutations to investigate and analyze the correlation between CCR6 and these 2 factors. In future studies, we will further explore the mechanism and impact of related chemokines, including CCR6, in LCH development. This study provides a reference for the diagnosis and targeted treatment of LCH.

The current first-line treatment for pediatric LCH involves a combination of vinblastine and prednisone acetate. The second-line treatment mainly involves cytarabine, and further rescue treatments may involve a combination of cladribine and cytarabine. However, as their efficacy improves, their toxicity and side effects also increase.^36–38^ Targeted therapy may reduce toxicity and side effects while improving efficacy. Based on the latest understanding of LCH pathogenesis, the current *BRAF* V600E inhibitor targeting the RAS signaling pathway has proven to be effective in the treatment of refractory and relapsed LCH. However, the follow-up time for most patients is less than 1 year, and long-term prognosis and adverse reactions cannot be determined.^39^ Notably, vemurafenib and dabrafenib target RAF, trametinib and cobimetinib target its downstream molecule MEK1/2, and the pipeline targets ERK1/2. However, research on the MAPK signaling pathway is limited to the preclinical level,^40^ and targeted therapies aimed at chemokines are currently in the development stage. The CCR6–CCL20 signaling pathway could be a potential therapeutic target for autoimmune diseases.^36,37^ Some inhibitors targeting CCR6 have also been studied in disease models, but not in LCH models.^41,42^ The findings of this study provide a theoretical basis for the study of CCR6 inhibitors in LCH disease models, promoting the development of LCH-targeted drugs.

In conclusion, this study clarified the clinical and pathological diagnostic processes in children with LCH using light microscopy, electron microscopy, and immunohistochemistry. By determining CCR6 expression in the inflammatory environment and detecting mutations in BRAF, ALK, and MAP2K1 in the MAPK signaling pathway, we can establish a precise clinical stratification system for LCH treatment. This will help identify children with poor prognosis as early as possible, thereby improving the efficacy and prognosis of LCH in children.

## ACKNOWLEDGMENTS

This work was supported by the Beijing Research Ward Excellence Program (BRWEP2024W102090104). Funders had no role in study design, data collection, and analysis, decision to publish, or preparation of the manuscript. We thank Dr. Yinshi Piao and Yuping Bai from the Department of Pathology, Beijing Tongren Hospital, for their secretary support.
